# ShenQi FuZheng Injection combined with chemotherapy in the treatment of colorectal cancer: A meta-analysis

**DOI:** 10.1371/journal.pone.0185254

**Published:** 2017-09-27

**Authors:** Rongzhong Xu, Liubing Lin, Yong Li, Yan Li

**Affiliations:** 1 Oncology Department of Shanghai Municipal Hospital of Traditional Chinese Medicine, Shanghai University of Traditional Chinese Medicine, Shanghai, China; 2 Digestive Department of Shanghai Municipal Hospital of Traditional Chinese Medicine, Shanghai University of Traditional Chinese Medicine, Shanghai, China; Stavanger University Hospital, NORWAY

## Abstract

**Objective:**

This study aims to investigate cellular immunity and clinical efficacy of ShenQi FuZheng Injection (SFI) in the associated chemotherapy of colorectal cancer (CRC).

**Methods:**

PubMed, Cochrane Library, EMBASE, China National Knowledge Infrastructure (CNKI), Chinese Scientific Journals Full-text Database (VIP), WanFang Database and Chinese Biomedical Literature Database (CBM) searches were undertaken to identify randomized controlled trials of SFI plus chemotherapy versus chemotherapy alone in CRC patients. The quality of each trial was assessed according to the Jadad’s scale, and Review Manager 5 was used to statisitically analyze the outcomes.

**Results:**

Eight studies involving 722 patients were included in this review. The meta-analyses suggested there was a significantly higher overall response rate (OR 1.89; CI: 1.10–3.24; p = 0.02), grades of KPS (OR 2.35; CI: 1.55–3.56; p<0.01), CD3+cells (MD 10.29; CI: 8.46–12.12; p<0.01), CD4+cells (MD 7.06; CI: 5.33–8.794; p<0.01), CD4/CD8+cells (MD 0.32; CI: 0.25–0.40; p<0.01), NK+ (MD 7.20; CI: 2.02–12.37, p = 0.006), WBC (MD 1.24; CI: 0.59–1.89; p<0.01), HB (MD 14.55; CI: 7.47–21.63; p<0.01), and PLT (MD 19.05; CI: 4.29–33.81; p = 0.01), but lower severe toxicity for leukocytopenia (OR 0.37; CI: 0.17–0.80; p = 0.01), thrombocytopenia (OR 0.32; CI: 0.14–0.74; p = 0.008), gastrointestinal toxicity (OR 0.48; CI: 0.24–0.96; p = 0.04), when chemotherapy combined with SFI was compared with chemotherapy alone. There were similarities between two groups in liver dysfunction (OR 0.44; CI: 0.18–1.08; p = 0.07) and CD8+ (MD 0.54; CI: -1.89–2.96; p = 0.66). Also, there was presence of heterogeneity in the CD8 results; after the sensitivity analysis, the result of CD8+ was reversed (MD 1.57; CI: 0.32–2.81; p = 0.01). There was no significant publication bias across studies according to the Egger’s (P = 0.19) and Begg’s test (P = 0.23).

**Conclusion:**

SFI enhances chemotherapy efficiency as they are combined and used in the treatment of colorectal cancer patients. At the same time, SFI also improves patients’ immunity function.

## Introduction

Colorectal cancer (CRC) is a malignant tumor in digestive tract worldwide, which accounts for about 9.0% of all cancer deaths [1,2]. In recent years, the incidence of colorectal cancer in western developed countries has decreased as nutrition structures changed [3,4]. However, the colorectal cancer incidence rises in China as more meat is chosen as the source of people’s nutrition. Surgery is the primary option for patients in early stages [5]. In contrast, the only treatment option for the patients in advanced stage is chemotherapy as liver metastasis has been found in nearly 25% of colorectal cancer patients during initial diagnosis [6]. Although chemotherapy has big advantages in both clinical efficiency and safety its side-effects, such as hematologic toxicity, myelosuppression and gastrointestinal toxicity still seriously disturb immune function and life quality of the patients [7].

Traditional Chinese medicine has become a promising alternative therapy for the treatment of colorectal cancer, because it has a unique advantage in reducing adverse reactions after radiotherapy, chemotherapy and surgery [8]. In China, the combination of traditional Chinese medicine with radiotherapy and chemotherapy has become a standard and important comprehensive treatment for colorectal cancer [9]. Some alkaloids extracted from traditional Chinese medicine have been widely used in clinic because of their low side effect and broad anti-tumor spectrum. ShenQi FuZheng Injection (SFI) is one kind of alkaloids which has been widely used in cancer clinical treatment in China. It is generally known that SFI, which contains two herbs—Codonopsis pilosula and Astragali is used extensively throughout China to modify the immunological function of malignant patients [10]. A systematic review on ShenQi FuZheng Injectionin 16 randomized controlled trials has reported a significant benefit in evaluating the curative effect on patients with advanced non small cell lung cancer (NSCLC) [11]. The results exhibited that chemotherapy combined with SFI could improve function of cellular immunity, prolong survival rate, and reduce toxicity. Currently, a number of published studies of SFI for treatment of colorectal cancer in combination with chemotherapy have shown that SFI could enhance total objective response rate, enhance the immunity, and reduce the toxicity of standard platinum-based chemotherapy. However, the efficacy and safety of SFI for the colorectal cancer patients have not been systemically reviewed by far.

In this review, we used meta-analysis to assess the efficacy, safety and immune-enhancement of SFI for treatment of colorectal cancer in combination with chemotherapy.

## Methods

### Inclusion criteria

Included studies must meet the following criteria: (1) Study population should be diagnosed and confirmed with colorectal cancer; (2) there are randomized controlled trials (RCTs); (3) interventions must be SFI combined with chemotherapy treatment; (4) comparison is made between intervention group and group of chemotherapy treatment alone; (5) one or more of the following outcomes are measured: effectiveness rates, performance status (the Karnofsky performance scale), blood system, immune function, and adverse events.

### Exclusion criteria

Excluded studies must meet the following criteria: (1) interventions were not the comparison between SFI combined with chemotherapy and chemotherapy alone in the treatment of colorectal cancer; (2) the language of references was not English or Chinese.

### Search strategy

The PubMed (1966 to August 2016), EMBASE (1974 to August 2016), Cochrane Library (1988 to August 2016), China National Knowledge Infrastructure Database (1979 to August 2016), WanFang Database (1990 to August 2016), Chinese Scientific Journals Full-Text Database (1989 to August 2016), and China Biological Medicine Database (1978 to August 2016) were searched for randomized controlled trials. The searching keywords contained: ShenQi FuZheng Injection, colorectal neoplasms, chemotherapy and multiple synonyms for each term. The languages were limited to Chinese and English.

### Data extraction and quality assessment

Two professional reviewers (RZX and LBL) independently extracted relevant data from texts, tables and figures. If the two investigators disagreed over a particular article, a third investigator would be consulted to reach a final consensus. The following information was recorded for each study: authors, year of publication, study aims, details of intervention, sample size, outcomes, the Jadad score, effectiveness rates, the Karnofsky performance scale, blood system, immune function, and adverse events. An open assessment of the trials was performed according to the seven-point Jadads cales [12]. This standard for evaluation is composed of randomization, allocation concealment, blinding as well as dropouts and withdrawals to assess the methodological quality for the 8 RCTs. Studies with scores of 0 to 3, 4 to 7 were evaluated as low and high quality, respectively. Only studies with a score of at least 3 were included in the analysis.

### Statistical analysis

In this study, statistical analysis was performed using software provided by the Review Manager 5 software as odds ratio (OR), mean difference (MD) and corresponding 95% confidence interval (CI). Both fixed-effect and random-effect models were used for meta-analysis. Despite the above two models showed similar outcomes, results from the random-effect model, which assume that the true underlying effect varies among selected studies, are expressed here [13,14]. The overall OR and 95% CI of objective tumor response, KPS score evaluation and adverse reactions were calculated using Mantel-Haenszel method. The overall weighted mean difference (WMD) and 95% CI of immune function and safety evaluation of blood system were also performed using Inverse-variance method. Significant heterogeneity was considered to be present for P≤ 0.1 in the Q test or for I^2^ > 50% [15]. Meanwhile, sensitivity analyses were conducted to explore the sources of heterogeneity. Funnel plots and Begg’s or Egger’s tests were created to detect publication bias, and P values <0.05 were considered statistically significant for all included studies.

## Results

### Search results

**Fig 1:**
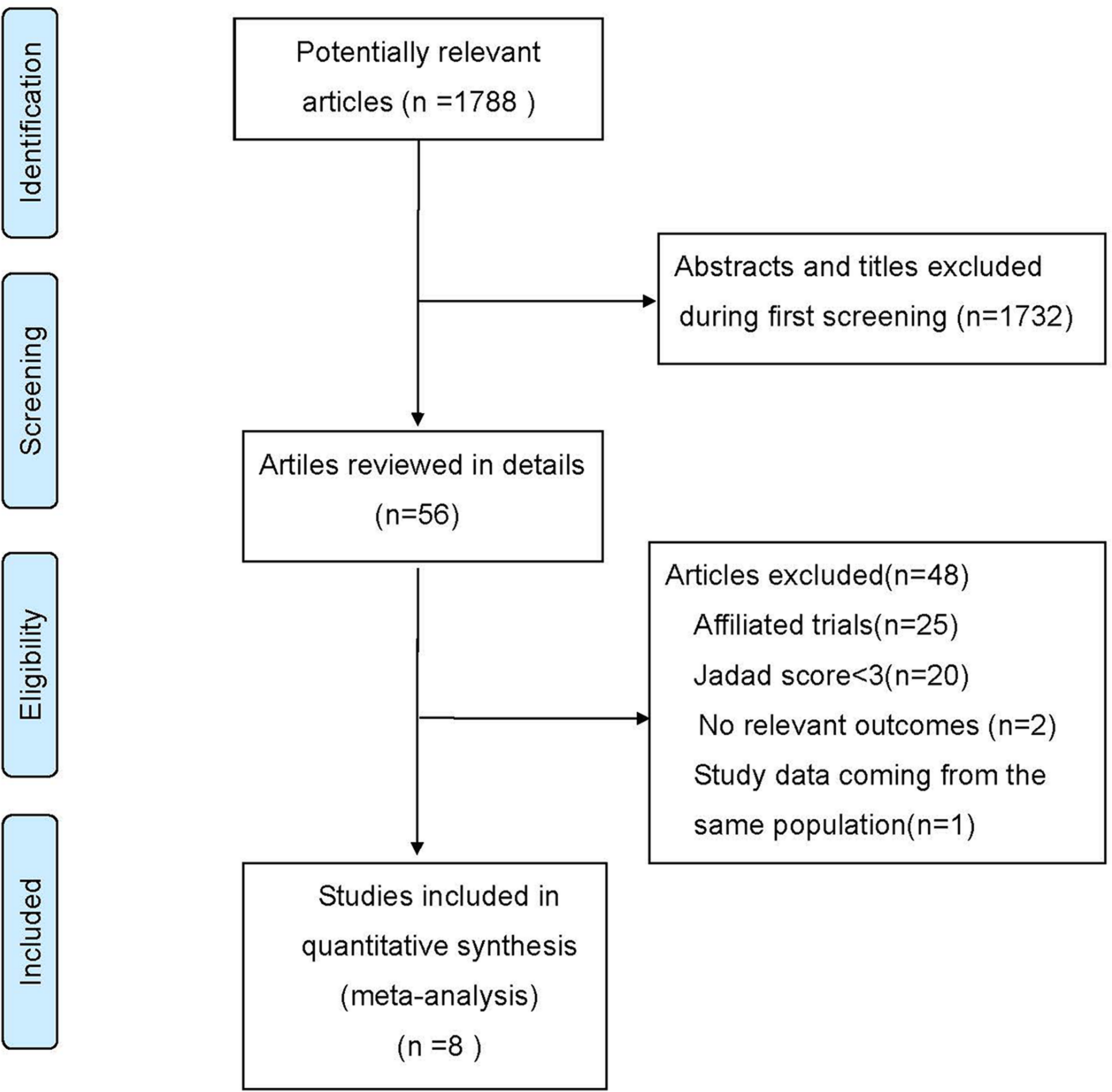
Flowchart of study selection procedure.

Fig 1 summarized the main details of the selected studies. We identified a total of 1788 articles from the initial literature search. After screening titles and abstracts, 56 publications were left during first screening. The reasons for exclusion of the other studies were duplication, non-RCT, patients not satisfying the inclusion criteria and the language not being English or Chinese. After reading the full text of the remaining 56 articles, we excluded 48 articles because they were affiliated trials, with Jadad scores<3 points, without relevant outcomes or they presented study data coming from the same population. Finally, a total of 8 trials were included for meta-analysis [16–23].

### Characteristics of studies

Eight eligible trials have been identified [16–23]. A total of 722 patients (experimental groups: 365 cases; the control groups: 357 cases) were included in this study. All studies were published from 2009 to 2015 and conducted in China. Seven of the trials used the SFI plus FOLFOX regimen [16–22]; one trial used SFI combined with XELOX regimen [23]. Two of the studies showed objective tumor response [16,23]; four reported KPS Score Evaluation [16,20–22]; seven showed outcomes of immune function [16,17,19–23]; two reported safety evaluation of blood system [17,21], and three elaborated on adverse reactions [18,19,22]. One trial had a Jadad score of 5 [16], three trials scored 4 [17–19], and four trials scored 3 [20–23]. The baseline characteristics in each trial are shown in Table 1.

**Table 1:** Basic characteristics of trials included in the study.

Studies	N (T/C)	Sex (M/F)	Age		KPS	Stage	Intervention		Type of Assessable Outcomes	Jadad scores
			T	C			T	C		
Liang QL 2009	76/76	101/51	NR		≥60	Ⅲ∼Ⅳ	FOLFOX+SFI (SFI250mL/d,d1-d10)	FOLFOX	KPS;CR,PR;CD4/CD8	5
Zhang XH 2009	40/36	NR	37~80	37~79	NR	NR	FOLFOX4+SFI (SFI250mL/d,d1-d7)	FOLFOX4	WBC,HB,PLT;CD4,CD8, CD4/CD8	4
Zhang Y 2010	20/20	23/17	35~61	35~63	NR	NR	FOLFOX+SFI (SFI250mL/d,d1-d5)	FOLFOX	gastrointestinal toxicity, liver dysfunction	4
Wang CB 2010	40/40	36/44	35~68	34~70	>60	Ⅲ	FOLFOX4+SFI (SFI250mL/d,d1-d5)	FOLFOX4	CD4,CD8,CD4/CD8;leukopenia, thrombocytopenia,gastrointestinal toxicity,liver dysfunction	4
Zuo JL 2012	45/44	49/40	27~91		>60	Ⅱ∼Ⅲ	FOLFOX4+SFI (SFI250mL/d,d1-d7)	FOLFOX4	KPS;CD3,CD4,CD8,CD4/CD8	3
Yan F 2014	56/56	68/44	36~83	36~84	>60	Ⅱ∼Ⅳ	FOLFOX4+SFI (SFI250mL/d,d1-d5)	FOLFOX4	KPS;WBC,HB,PLT;CD3,CD4, CD8,CD4/CD8;	3
Song M 2015	45/44	52/37		51~73	>60	Ⅱ∼Ⅲ	FOLFOX4+SFI (SFI250mL/d,d1-d14)	FOLFOX4	KPS;CD3,CD4,CD8,CD4/CD8; Leukopenia,thrombocytopenia, gastrointestinal toxicity, liver dysfunction	3
Zhang W 2015	43/43	57/29	51~72	52~73	≥60	Ⅲ∼Ⅳ	XELOX+SFI (SFI250mL/d,d1-d14)	XELOX	KPS;CR,PR;CD4,CD8,CD4/CD8,	3

T/C: Experimental group/control group; NR: Not reported; D: Day; SFI: ShenQi FuZheng Injection; FOLFOX: L-OHP and Calcium Folinate and 5-Fu; XELOX: L-OHP and sanofi-aventis and capecitabine; KPS: Karnofsky; CR: Complete response; PR: Partial response; WBC: White blood cell; PLT: Platelet; HB: Hemoglobin.

### Effectiveness

**Fig 2:**
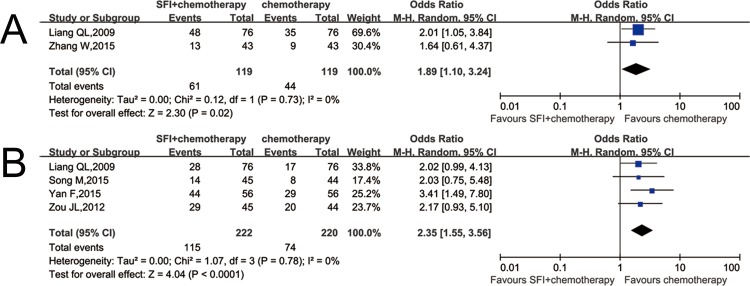
Comparison of efficacy and KPS between SFI/chemotherapy and chemotherapy. A: The efficacy increased when using SFI; B: KPS scores increased when using SFI.

Two studies that include 238 patients described the objective tumor response [16,23]. Statistical differences in the two groups were found in the proportion of patients who achieved overall response rate (ORR, or complete response plus partial response; OR 1.89; CI: 1.10–3.24, p = 0.02; I^2^ = 0%) (Fig 2A). This result revealed a higher overall response rate in experimental group than that in the control group, which declared that SFI plus chemotherapy can significantly improve the efficiency of clinical curative effect on patients when compared with chemotherapy alone.

### KPS score evaluation

Four RCTs containing 442 patients reported KPS [16,20–22]. The result showed that there is a statistically significant difference between two groups, which means that SFI combined with chemotherapy had better KPS score than chemotherapy alone, further to improve quality of life when compared with chemotherapy alone (OR 2.35; CI: 1.55–3.56, p<0.01; I^2^ = 0%) (Fig 2B).

### Immune function

**Fig 3:**
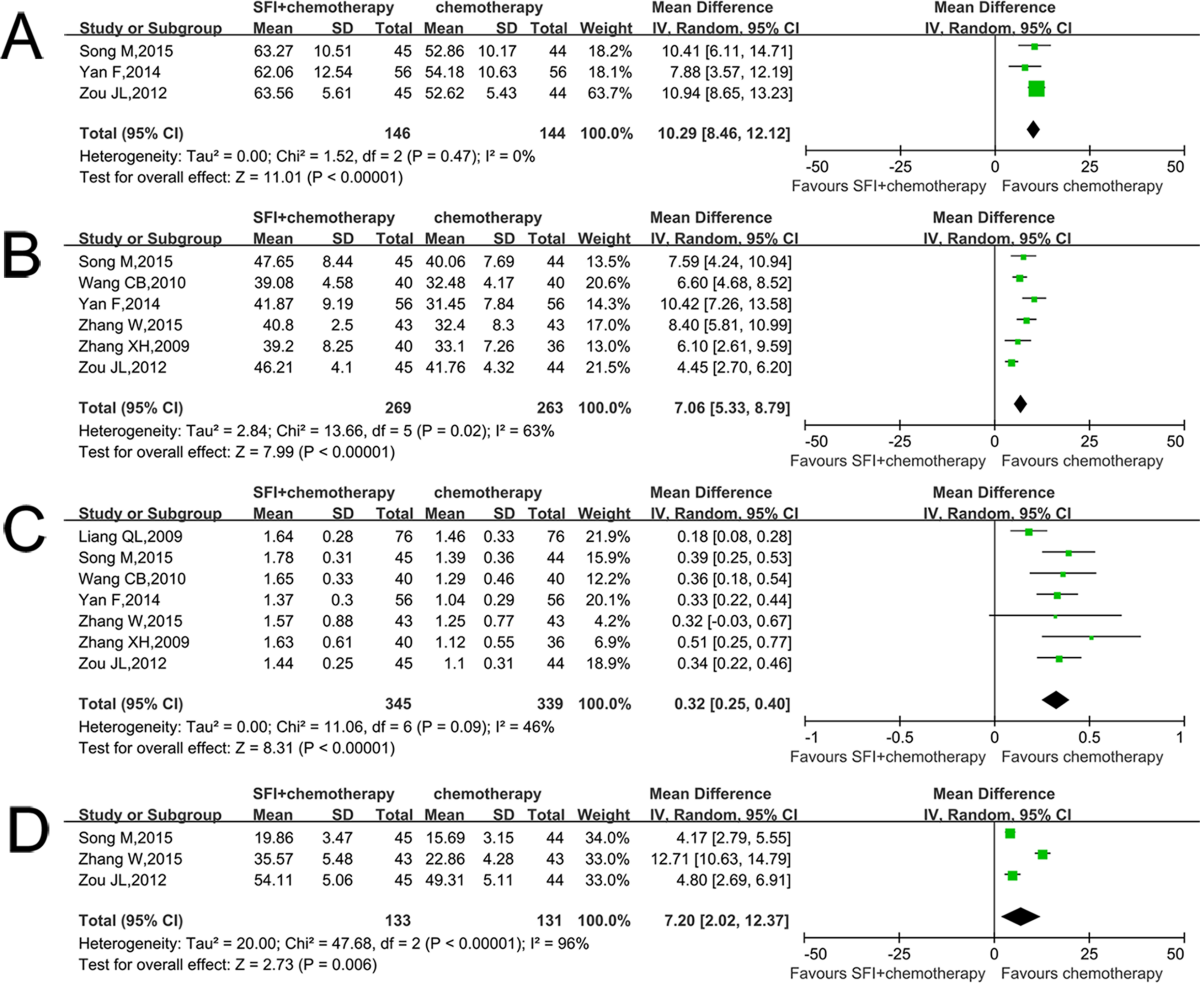
Comparison of immune function between SFI/chemotherapy and chemotherapy. A: The percentage of CD3+ increased when using SFI; B: The percentage of CD4+ increased when using SFI; C: The percentage of CD4/8 increased when using SFI; D: The percentage of NK increased when using SFI.

The CD3+ expression was reported in 3 trials including 290 patients [20–22]. The patients treated with combined therapy had a higher MD than those treated with chemotherapy alone (MD 10.29; CI: 8.46–12.12, p<0.01; I^2^ = 0%) (Fig 3A). According to this result, meta-analysis revealed that SFI plus chemotherapy can increase the CD3+ expression in patients.

Six RCTs of the 8 studies containing 532 patients reported the CD4+ expression [17,19–23]. Meta-analysis showed that the patients treated with combined therapy had a higher MD than those treated with chemotherapy alone (MD 7.06; CI: 5.33–8.79, p<0.01; I^2^ = 63%) (Fig 3B), which explains that SFI plus chemotherapy can significantly increase the percentage of CD4+ expression. There was considerable heterogeneity among the included trials (P = 0.02). After Zou JL’s study was excluded [20], the heterogeneity test showed P = 0.27, indicating that there was no statistical heterogeneity between studies, but the conclusion was not affected (MD 7.69; CI: 6.28–9.10, p = 0.02; I^2^ = 23%).

Seven trials including 684 patients provided data regarding CD4+/CD8+ expression [16,17,19–23]. The results illustrated that the patients treated with combined therapy had a higher MD than those treated with chemotherapy alone (MD 0.32; CI: 0.25–0.40, p<0.01; I^2^ = 46%) (Fig 3C), which explains that SFI combined with chemotherapy in the treatment of colorectal cancer can significantly increase extent of the CD4+/CD8+ expression.

Three trials containing 264 patients that provided NK+ results showed that the patients treated with combined therapy had a higher MD than those treated with chemotherapy alone (MD 7.20; CI: 2.02–12.37, p = 0.006; I^2^ = 96%) (Fig 3D), which indicates that SFI combined with chemotherapy in the treatment of colorectal cancer can significantly improve the level of the NK+ expression [20,22,23]. After Zhang W’s study was excluded, the heterogeneity test showed P = 0.624, indicating that there was no statistical heterogeneity between studies, but the conclusion was not affected (MD 4.36; CI: 3.20–5.51, p<0.01; I^2^ = 0%) [23].

**Fig 4:**
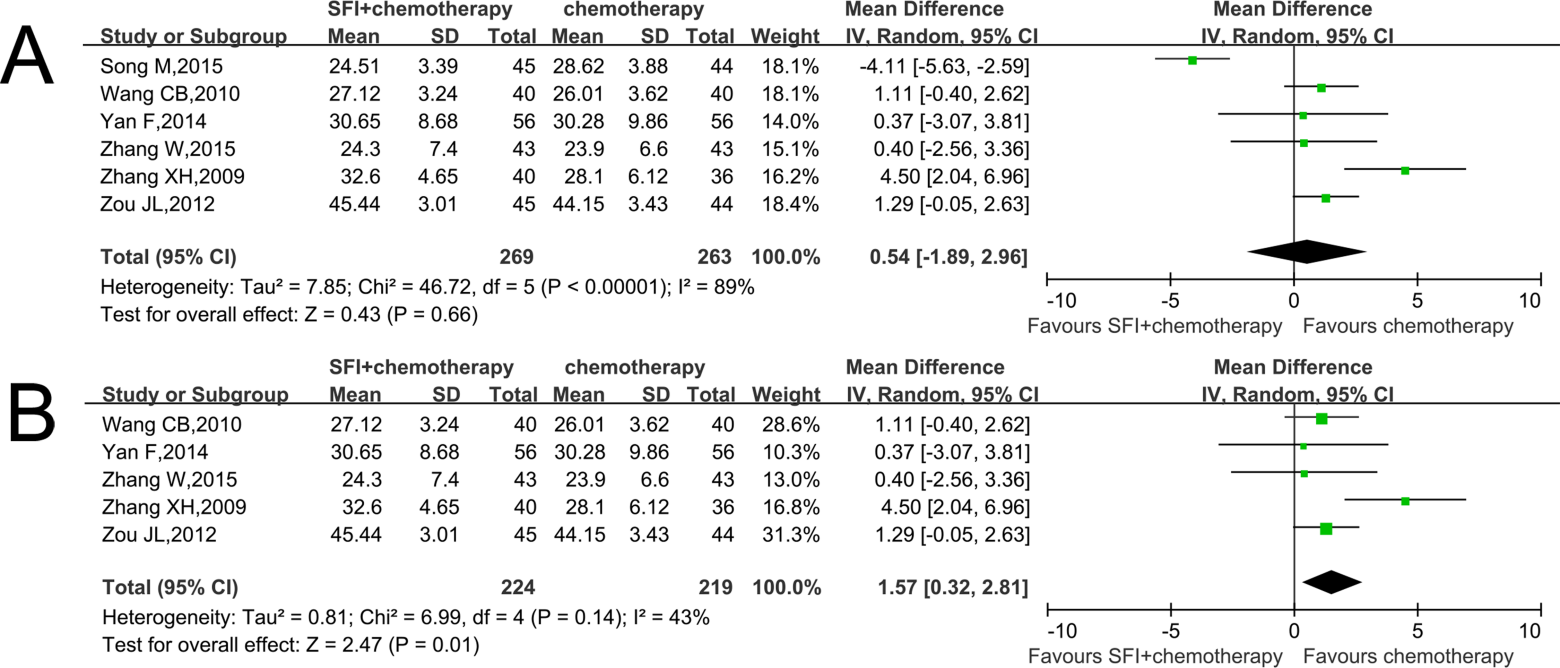
Comparison of CD8+ between SFI/chemotherapy and chemotherapy. A: The percentage of CD8+ increased when using SFI; B: Sensitivity analysis was performed by omitting one study.

Six RCTs including 532 patients provided data relevant to analyzing the CD8+ expression [17,19–23]. This result indicated that there is no statistical difference between two groups (MD 0.54; CI: -1.89–2.96, p = 0.66; I^2^ = 89%) (Fig 4A). As there was significant heterogeneity, we conducted a sensitivity analysis. This result was reversed when Song M’s study was excluded [22]. The results showed that the patients treated with combined therapy had a higher MD than those treated with chemotherapy alone (MD 1.57; CI: 0.32–2.81, p = 0.01; I^2^ = 43%) (Fig 4B), which explains that SFI combined with chemotherapy in the treatment of colorectal cancer had an advantage of increasing the percentage of helper CD8+ compared with control group.

### Safety evaluation of blood system

**Fig 5:**
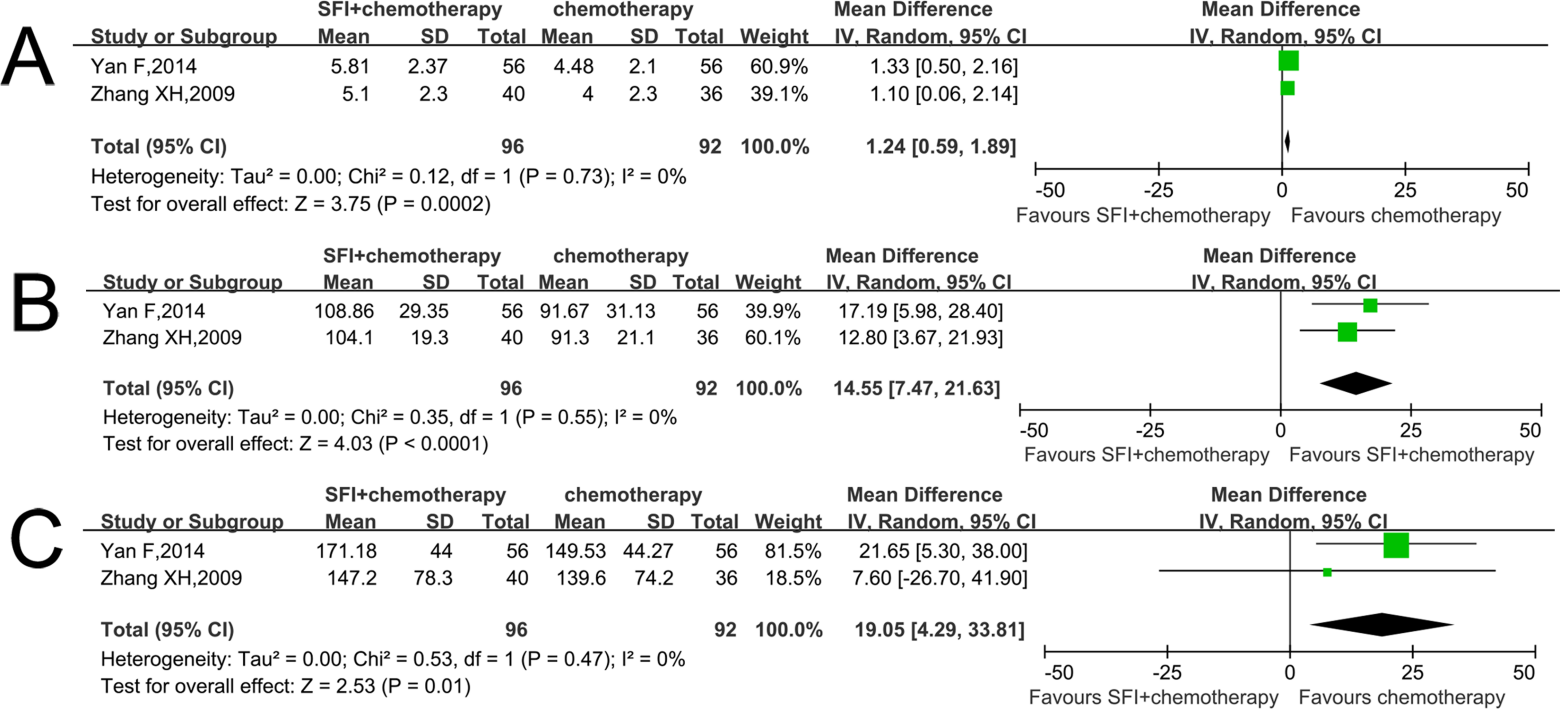
Comparison of blood system between SFI/chemotherapy and chemotherapy. A: WBCs increased when using SFI; B: HBs increased when using SFI; C: PLTs increased when using SFI.

Two RCTs reported safety evaluation of WBCs, HBs and PLTs [17,21]. The MDs of WBCs, HBs and PLTs were 1.24 (CI: 0.59–1.89, p<0.01; I^2^ = 0%) (Fig 5A), 14.55 (CI: 7.47–21.63; p<0.01, I^2^ = 0%) (Fig 5B), 19.05 (CI: 4.29–33.81; p = 0.01, I^2^ = 0%) (Fig 5C), respectively. This result indicated that WBCs, HBs and PLTs in the test group where patients were treated with combined therapy were significantly higher than those treated with chemotherapy alone.

### Toxicity

**Fig 6:**
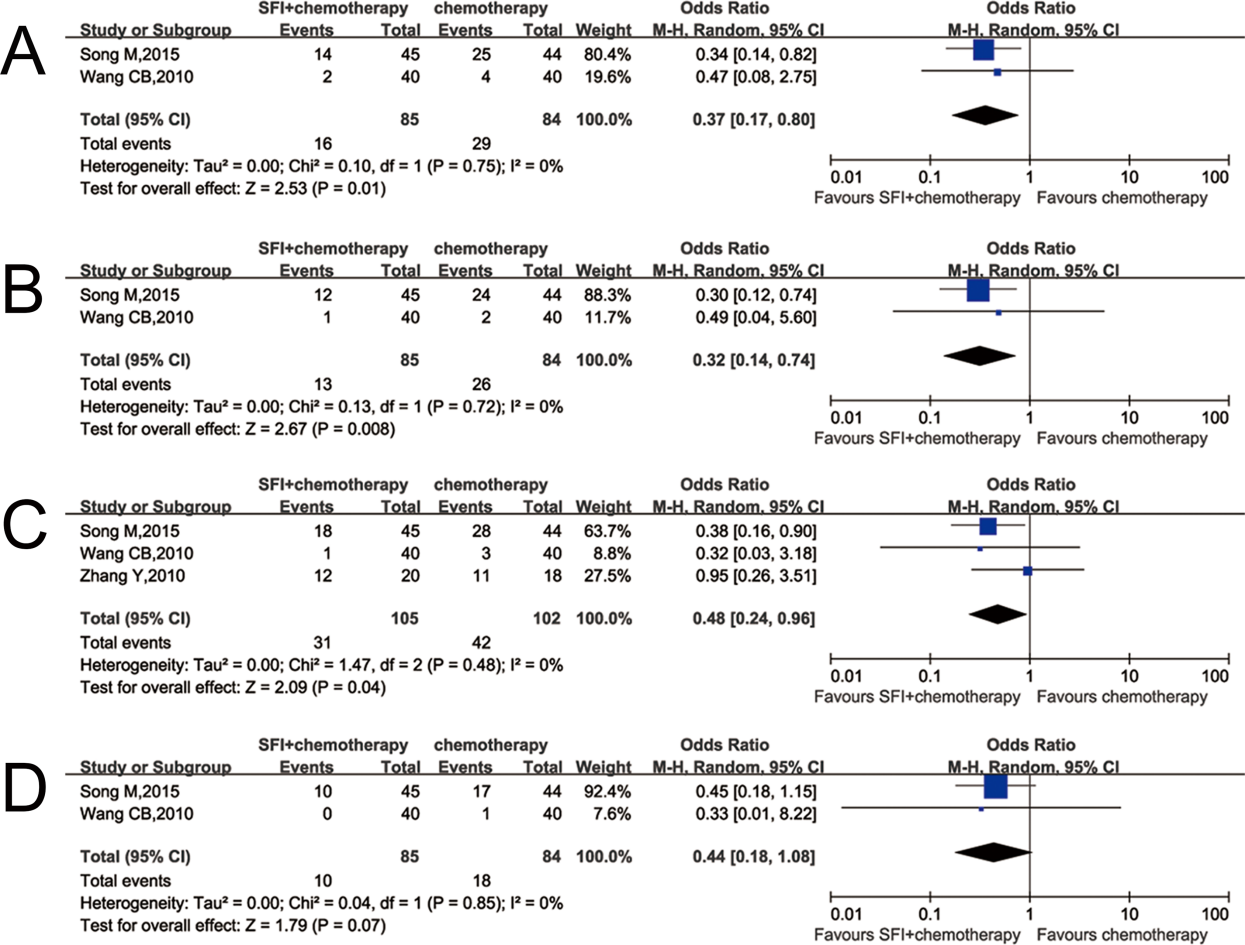
Comparison of adverse events between SFI/chemotherapy and chemotherapy. A: Leukocytopenia; B: Thrombocytopenia; C: Gastrointestinal toxicity; D: Liver dysfunction.

Two trials [19,22] provided the results of hematological toxicity and liver dysfunction and three [18,19,22] reported gastrointestinal toxicity. The ORs of leukocytopenia, thrombocytopenia, gastrointestinal toxicity and liver dysfunction were 0.37 (CI: 0.17–0.80, p = 0.01; I^2^ = 0%) (Fig 6A), 0.32 (CI: 0.14–0.74, p = 0.008; I^2^ = 0%) (Fig 6B), 0.48 (CI: 0.24–0.96, p = 0.04; I^2^ = 0%) (Fig 6C), 0.44 (CI: 0.18–1.08, p = 0.07; I^2^ = 0%) (Fig 6D), respectively. This result indicated patients treated with SFI plus chemotherapy have a decreasing risk of leukocytopenia, thrombocytopenia and gastrointestinal toxicity compared with the chemotherapy control group. There were similarities between two groups in liver dysfunction when it is suggested that SFI plus chemotherapy in the treatment of colorectal cancer fails to reduce the damaging incidence of liver when compared with chemotherapy alone.

### Publication bias

**Fig 7:**
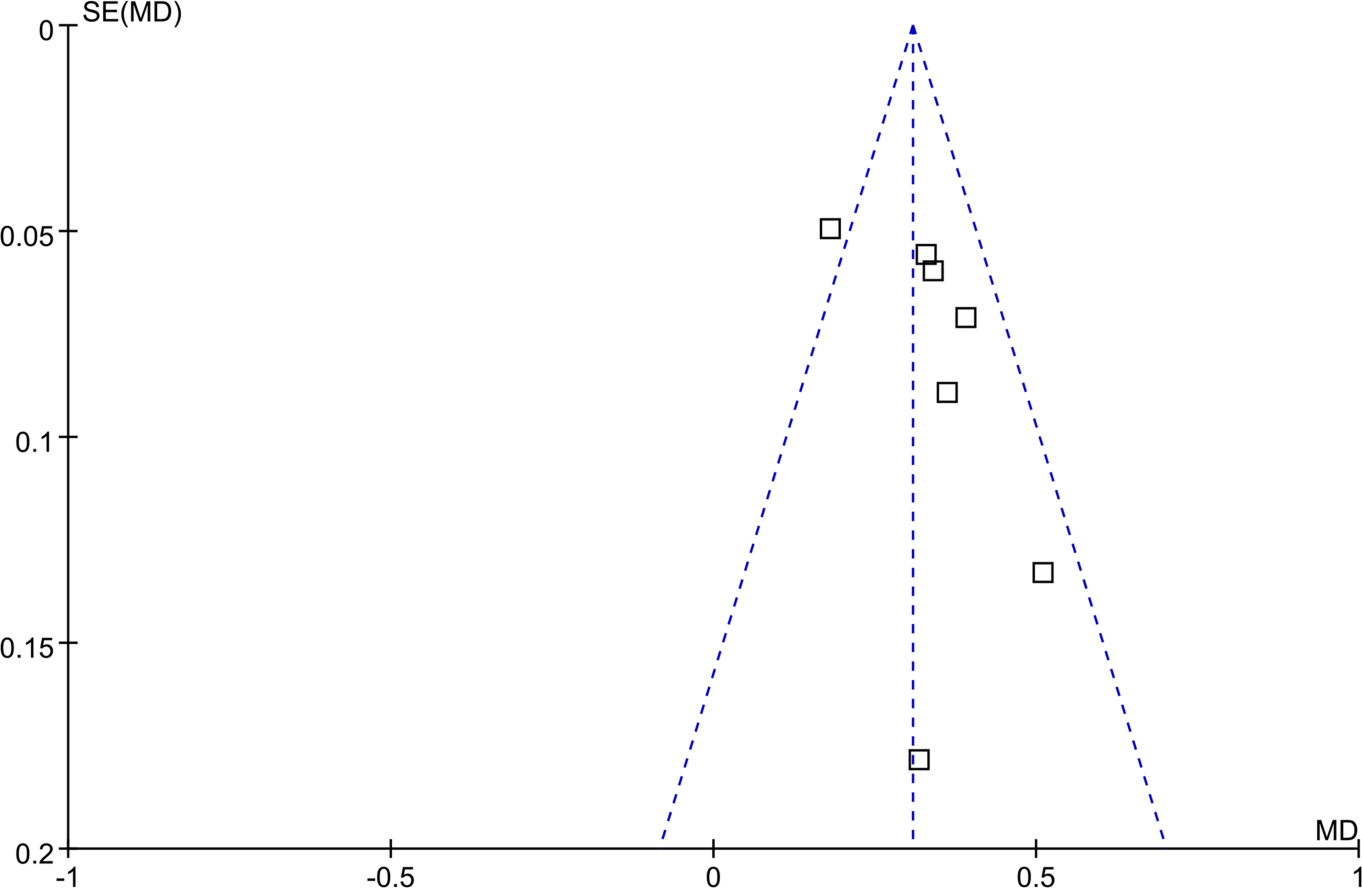
Funnel plot, based on studies with data on CD4+/CD8+.

Since the majority of the articles included were reporting immune index, funnel plots based on the data for MD of CD4/CD8 were elaborated to evaluate publication bias on Fig 7. We did not identify any significant graphic and statistical bias according to the Egger’s (P = 0.19) and Begg’s test (P = 0.23).

## Discussion

SFI comprises of Codonopsis pilosula and Astragali, which have been used to improve immune functions to fend off non-small-cell lung cancer, cancer of the stomach and hepatocarcinoma, etc [9]. Astragalus increases the secretion of interferon and tumor necrosis factor, and activates lymphocytes, natural killer (NK) cells and macrophages against tumor [24–26]. Codonopsis pilosula also has the effects of anti-tumor, antimicrobial, anti-oxidation, and improvement of cellular immunity [27–29]. Codonopsis pilosula inhibits the tumor weight in vivo, stimulates splenocytes proliferation, enhances the macrophages phagocytosis and improves the NO production in macrophages [30]. A total of 8 trials were considered eligible for the meta-analysis reporting SFI plus chemotherapy versus chemotherapy alone in treating colorectal cancer. And the meta-assay results suggested that SFI intervention improves the clinical effect and the quality of survival (KPS), strengthens cellular immune function (CD3+, CD4+, CD4+/CD8+ and NK+), and reduces the adverse events such as leukocytopenia, thrombocytopenia and gastrointestinal toxicity. In addition, no significant difference was observed between two groups in CD8+ and liver dysfunction. As there was a significant heterogeneity in CD8+, we conducted the sensitivity analysis. Sensitivity analysis revealed that Song M’s study is the source of statistical heterogeneity in meta-analysis for the outcome of CD8+. This result was reversed when the Song M’s study was excluded [17] and meta-analysis of these five trials indicated that SFI combined with chemotherapy in the treatment of colorectal cancer had an advantage of increasing the percentage of helper CD8+ compared with control group. Because the result of CD8+ is not stable, we were unable to give a definite conclusion. Therefore, the results of CD8+ need to be proved by higher quality trials and larger samples in the future.

We did not identify any significant graphic and statistical bias according to the Egger’s and Begg’s test of funnel plot. But this meta-analysis also has several limitations and shortcomings. Despite we searched PubMed, EMBASE and the Cochrane Library, all of the included studies were Chinese. The major limitations are poor quality of the included studies in our meta-analysis. Although all trials have performed randomization and reported the method of a table of random digits to generate the allocation sequence, no study accounted for double-blind, only one provided the methods of allocation concealment and two mentioned the withdrawals/dropouts. All trials were conducted only in single centre. In addition, a small sample size with 722 patients may lack statistical power to confirm the conclusion, which influences our findings to some extent. Although these shortcomings, this study still provides useful information for clinical practice and drug development to support the advantage of SFI treatment.

## Conclusion

SFI combined with chemotherapy in the treatment of colorectal cancer may improve the chemotherapy efficacy, enhance the immunity function and reduce the toxicity. However, considering the limited number of RCTs and the poor quality among the included studies, the results need to be further verified by high quality trials and large samples.

## Supporting information

S1 Table The 48 full-text excluded articles with reasons. (DOCX)

S1 File The PRISMA 2009 checklist. (DOC)

S2 File Search strategy. (DOC)

S3 File The available data. (XLS)
