# Supplementary material for: ShenQi FuZheng Injection combined with chemotherapy in the treatment of colorectal cancer: A meta-analysis
Source: PLoS One. 2017 Sep 27;12(9):e0185254. doi: 10.1371/journal.pone.0185254 (PMC5617195; doi:10.1371/journal.pone.0185254)
Supplement: S1 Table — (DOCX) [file pone.0185254.s001.docx]

**S1 Table The 48 full-text excluded articles with reasons**

| Excluded articles | Reasons |
| --- | --- |
| ZhangY, et al.[[1](#_ENREF_1)] | Study data coming from the same population |
| Sun Shuxian,et al.[[2](#_ENREF_2)] | No relevant outcomes |
| Gai Juanjuan, et al.[[3](#_ENREF_3)] | No relevant outcomes |
| Wang kun, et al.[[4](#_ENREF_4)] | Jadad score<3 |
| Ju Dongyang, et al. [[5](#_ENREF_5)] | Jadad score<3 |
| MIAO Ke, et al.[[6](#_ENREF_6)] | Jadad score<3 |
| Jiang Chunqi, et al.[[7](#_ENREF_7)] | Jadad score<3 |
| Ni Bingqiang, et al.[[8](#_ENREF_8)] | Jadad score<3 |
| Tan Guanggen,et al.[[9](#_ENREF_9)] | Jadad score<3 |
| Dai Chuang, et al.[[10](#_ENREF_10)] | Jadad score<3 |
| Wang Zhenhai, et al.[[11](#_ENREF_11)] | Jadad score<3 |
| Xing Feng, et al.[[12](#_ENREF_12)] | Jadad score<3 |
| Peng Ying, et al.[[13](#_ENREF_12)] | Jadad score<3 |
| Zhang Yan, et al.[1[4](#_ENREF_4)] | Jadad score<3 |
| HuoWei, et al. [1[5](#_ENREF_5)] | Jadad score<3 |
| LI Shi Dong, et al.[1[6](#_ENREF_6)] | Jadad score<3 |
| Liu Min, et al.[1[7](#_ENREF_7)] | Jadad score<3 |
| Luo Zheng, et al.[1[8](#_ENREF_8)] | Jadad score<3 |
| Jia Chunying, et al.[1[9](#_ENREF_9)] | Jadad score<3 |
| Jin Xinrong, et al.[20] | Jadad score<3 |
| Guo Yarong, et al.[[21](#_ENREF_11)] | Jadad score<3 |
| Zhao Tong, et al.[[22](#_ENREF_12)] | Jadad score<3 |
| Jia Youpeng, et al.[[23](#_ENREF_12)] | Jadad score<3 |
| Han Zhuoyue, et al.[[24](#_ENREF_12)] | Affiliated trials |
| Wang Yuling, et al.[[25](#_ENREF_12)] | Affiliated trials |
| Gao Tong, et al.[[26](#_ENREF_12)] | Affiliated trials |
| Zhang Binggui, et al.[[27](#_ENREF_1)] | Affiliated trials |
| Chen Huajun, et al.[[2](#_ENREF_2)8] | Affiliated trials |
| Wang Wei, et al.[[2](#_ENREF_2)9] | Affiliated trials |
| Kang Hanwen, et al.[[30](#_ENREF_2)] | Affiliated trials |
| Luo Dong, et al.[[31](#_ENREF_2)] | Affiliated trials |
| Qian Meifen, et al.[[32](#_ENREF_1)] | Affiliated trials |
| Xu Xiaoyong, et al.[[33](#_ENREF_2)] | Affiliated trials |
| Liu Zifeng, et al.[34] | Affiliated trials |
| Ni Bingqiang, et al.[[35](#_ENREF_2)] | Affiliated trials |
| Huang Zhuohua, et al.[36] | Affiliated trials |
| Zhu Heting, et al.[[37](#_ENREF_1)] | Affiliated trials |
| Meng Yuan, et al.[[38](#_ENREF_2)] | Affiliated trials |
| Zhao Jun, et al.[39] | Affiliated trials |
| Wang weihua, et al.[40] | Affiliated trials |
| Jing Xinrong, et al.[41] | Affiliated trials |
| Xin Ming, et al.[42] | Affiliated trials |
| Li Naiqing, et al.[[43](#_ENREF_2)] | Affiliated trials |
| ChengHuijun, et al.[[44](#_ENREF_2)] | Affiliated trials |
| Sun Daxing, et al.[[45](#_ENREF_2)] | Affiliated trials |
| Zhu Xia, et al.[[46](#_ENREF_2)] | Affiliated trials |
| Yang Yan, et al.[47] | Affiliated trials |
| Lu Dehong, et al.[[48](#_ENREF_2)] | Affiliated trials |

**References**

1. Zhang Yan, Xia Zhimin, Tan Yanwei, Yang Xitang. Influence of ShenQi FuZheng Injection combined with chemotherapy on body immune function. Modern Journal of integrated traditional Chinese and Western medicine. 2011,(22):2735-2737.
2. Sun Shuxian. Effects of ShenQi FuZheng Injection on life quality of patients with coloncancer. Journal of Hainan Medical University. 2012,(10):1438-1440.
3. Gai Juanjuan. Effect of ShenQi FuZheng Injection on postoperative CapeOX chemotherapy in patients with stage Ⅱ/Ⅲ Clinical study on the influence of patients' quality of life. Journal of practical medicine. 2015,(06):1014-1016.
4. Wang kun, Tan Jianxiong, Long Yun. Clinical observation of ShenQi FuZheng Injection Combined with chemotherapy in the treatment of 30 cases of colorectal cancer. Yunnan Journal of traditional Chinese medicine. 2007,(04):20-21+63. DOI: 10.3969/j.issn.1007-2349.2007.04.017.
5. Ju Dongyang, Zhang Ruyi, Zhen Yunhuan, Ji Qinghua.Clinical Observation on Effect of ShenQi FuZhengInjection Combined with Chemotherapy in Treating post-operational patients with colorectal carcinoma. Chinese Journal of Integrated Traditional and Western Medicine. 2003,(03):228-230.
6. Miao Ke, Gong An-an. Effect of ShenQi FuZheng injection on serum CA19-9，CRP and CD4 + lymphocyte subsets in patients after laparoscopic radical resection of colon cancer. Chinese Journal of biochemical medicine. 2015,(10):51-53.
7. Jiang Chunqi, Shen Jieru, Liu Wenqi, Huang Liming. Effect of ShenQi FuZheng Injection on immune function of patients with colon cancer after chemotherapy. Heilongjiang traditional Chinese medicine. 2015,(02):41-42.
8. Ni Bingqiang, Zhang Zhihong, Chen Rixin, Luo Zhanxiong. Clinical study on the effect of ShenQi FuZheng Injection on postoperative chemotherapy of colorectal cancer. Guangxi medical journal. 2009,(05):644-646. DOI: 10.3969/j.issn.0253-4304.2009.05.016.
9. Tan Guanggen, Liu Li, Li Jing, Hu Yuezhen. Effects of ShenQi FuZheng Injection on immunologic function of patients with colon cancer. Journal of Hainan Medical University. 2013,(05):627-629.
10. Dai Chuang. Effect of ShenQi FuZheng Injection combined with chemotherapy in the treatment of colorectal adenocarcinoma. Strait pharmaceutical. 2012,(09):148-150. DOI:10.3969/j.issn.1006-3765.2012.09.078.
11. Wang Zhenhai. Effect of ShenQi FuZheng Injection on toxic and side effects of chemotherapy in patients with colon cancer. International Journal of traditional Chinese medicine (TCM). 2009,31(5):431-432. DOI: 10.3760/cma.j.issn. 1673-4246.2009.05.030.
12. Xing Feng, Feng Guo Ping. ShenQi FuZheng Injection combined with FOLFOX4 regimen in the treatment of advanced colorectal cancer. Chinese Journal of higher medical education. 2015,(1):140144. DOI: 10.3969/j.issn.1002-1701.2015.01.075.
13. Peng Ying, Tang Jianpo, Peng Yi. Study on the effect of ShenQi FuZheng decoction combined with chemotherapy in the treatment of advanced colon cancer. Henan traditional Chinese medicine. 2014,(07):1336-1337.

## Zhang Yan, Guo Linli, Li Hongyan. Clinical Study on the Efficiency of ShenQi FuZheng Injection Combined with Chemotherapy in the Treatment of Colorectal Carcinoma. China pharmacy. 2010,(04):357-359.

## HuoWei, LI Zhimin, Pan Xinyan. Observation of efficacy of ShenQi FuZheng Injection combined with chemotherapy intreatment of advanced colorectal cancer. Chinese Journal of clinical oncology and rehabilitation. 2008,(05):454-456.

## Li Shi Dong,Lv Rui,Wang Jiangtao,et al. Clinical observation of ShenQi FuZheng Injection and capectitabine in treatment of elderly patients with advanced carcinoma of large intestine. Chinese Minkang medical. 2014,(7):6-7,9.DOI:10.3969/j.issn.1672-0369.2014.07.003.

## Liu Min. Clinical study of ShenQi FuZheng Injection combined with irinotecan and cetuximab in treatment of advanced colorectal cancer. modern medicine and clinic. 2015, (10):1255-1258. DOI: 10.7501/j.issn.1674-5515.2015.10.017.

## Luo Zheng, Wei Xia, Xiong Zhanghua. The clinical study of Chinese medicine in rectal cancer protective effect of postoperative radiotherapy. new Chinese medicine. 2010,(09):37-39.

## Jia Chunying, Chang Shengli, Ma Liming, Qin Zhili. Clinical Effect and Impact on the Levels of Treg, TNF-α and IL-12 of ShenQi FuZheng Injection Combined with FOLFOX4 for advanced colorectal cancer. marker immunoassay and clinic. 2016,(04):382-384+388.

## Jin Xinrong, Chen Jian, Min min, Zhong Guocheng, et al.Clinical efficacy of ShenQi FuZheng Injection Combined with chemotherapy on advanced colorectal carcinoma. Modern Oncology. 2007,15(3):384-386. DOI: 10.3969/j.issn.1672-4992.2007.03.036.

## Guo Yarong, Jia Junmei, Zhao Heping,Chai Bao. ShenQi FuZheng Injection Combined with oxaliplatin combined with 5- fluorouracil treatment of 30 cases of advanced colorectal cancer with calcium leucovorin. Chinese Journal of medicine and clinical medicine. 2011,11 (2):221-223. DOI: 10.3969/j.issn.1671-2560.2011.02.055.

## Zhao Tong, Liu Yong. Clinical observation of ShenQi FuZheng Injection Combined with mFOLFOX6 regimen in treatment of advanced colorectal carcinoma. Chinese Journal of modern drug application. 2011,05 (5):16-17. DOI: 10.3969/j.issn.1673-9523.2011.05.009.

1. Jia Youpeng. Study on the effect of TNF- and CK20 on the invasion and metastasis of colon cancer and the effect of ShenQi FuZheng Injection. Dalian Medical University. 2008
2. Han Zhuoyue, Dai Jingyou, Zhang Xinchen. Application of ShenQi FuZheng Injection in elderly patients with rectal cancer after operation. Chinese Journal of traditional Chinese medicine information. 2012,(03):82-83. DOI : 10. 3969/j.issn.1005-5304.2012.03.037.
3. Wang Yuling, Gao Yuan, Yin Hong, Wu LingZhi. Effect of ShenQi FuZheng Injection on immune function of patients with malignant gastrointestinal tumor.traditional Chinese medicine and clinical pharmacology. 2008,(06):506-507. DOI: 10.3321/j.issn:1003-9783. 2008.06.029.
4. Gao Tong, Xue Yinping, Wang Xiaoyan, et al. Clinical observation on the effect of ShenQi FuZheng Injection on the side effects of chemotherapy in the elderly patients with advanced gastrointestinal cancer. medical information. 2011,(07):3253-3254. DOI: 10. 3969/j.issn.1006-1959.2011.07.447.
5. Zhang Binggui, Liu Kun, Liu Zhang, et al. Effect of ShenQi FuZheng Injection on nutritional and immune status in elderly patients with gastrointestinal tumor. tumor research and clinic. 2009,21(9):623-624. DOI:10.3760/cma.j.issn.1006-9801. 2009.09.017.
6. Chen Huajun, Xie Xinmei. Effect of ShenQi FuZheng Injection on immune function in patients with advanced malignant gastrointestinal tumors. Journal of modern medicine and hygiene. 2012,(16):2448-2449.
7. Wang Wei. Effect of ShenQi FuZheng Injection on immune function of postoperative patients with gastric cancer. contemporary medicine. 2010,(32):150. doi: 10.3969/j.issn. 1009-4393.2010.32.113.
8. Kang Hanwen. Shenqi Fuzheng Injection in adjuvant chemotherapy for gastrointestinal cancer. occupational health and disease. 2005,(01):75-76. DOI: 10.3969/j.issn. 1006-172X. 2005.01.060.
9. Luo Dong, Zhou Dingming. Analysis on the clinical efficacy of ShenQi FuZheng Injection auxiliary in treatment of middle-late gastrointestinal malignant tumor. Chinese Journal of biochemical medicine. 2014,(05):152-153+156.
10. Qian Meifen. Clinical observation on 48 cases of digestive tract tumor treated with ShenQi FuZheng Injection Combined with chemotherapy. Heilongjiang traditional Chinese medicine. 2008,(06):11-12.
11. Xu Xiaoyong. ShenQi FuZheng Injection Combined with chemotherapy in the treatment of advanced gastrointestinal cancer. Zhejiang Journal of integrated traditional Chinese and Western medicine. 2007,(07):412-413. DOI: 10.3969/j. issn.1005-4561.2007.07.009.
12. Liu Zifeng, Li Guixin, Ma Changgeng, Sun Xiumei. Study on the effect of ShenQi FuZheng Injection on cachexia of digestive tract tumor. Chinese Journal of modern drug application. 2012,(10):94-95.
13. Ni Bingqiang, Zhang Zhihong, Luo Zhanxiong, et al. ShenQi FuZheng Injection Combined with intraperitoneal hyperthermic perfusion chemotherapy in the treatment of advanced abdominal tumor. Chinese medical journal. 2008,(01):50-52. DOI:10.3969/j.issn. 1008-1070.2008.01.022.
14. Huang Zhuohua. Clinical effect of ShenQi FuZheng Injection combined with chemotherapy in treating advanced gastrointestine carcinoma. Chinese Journal of contemporary medicine. 2015,(35):61-63.
15. Zhu Heting, Lengkai. Clinical observation of ShenQi FuZheng Injection Combined with chemotherapy in the treatment of advanced gastrointestinal cancer. Chinese medical journal. 2003,(07):53. DOI: 10.3969/j.issn.1008-1070.2003.07.026.
16. Meng Yuan, Xiao Qiming, Guo Guigang, et al. Clinical observation of ShenQi FuZheng Injection Combined with chemotherapy in the treatment of gastric and colonic tumors. International Journal of traditional Chinese medicine. 2013,(9):828-829. DOI: 10. 3760/cma.j.issn.1673-4246.2013.09.019.
17. Zhao Jun. Clinical observation on treatment of 45 cases of digestive tract tumor with ShenQi FuZheng Injection Combined with chemotherapy. cancer progression. 2009,(05): 583-584.DOI: 10.3969/j.issn.1672-1535.2009.05.025.
18. Wang weihua. Clinical observation of ShenQi FuZheng Injection Combined with chemotherapy in the treatment of advanced gastrointestinal cancer.Strait Pharm J. 2013,25(1):113-114.doi: 10.3969/j.issn.1006-3765.2013.01.055.
19. Jing Xinrong, Chen Jian, Min Min, et al. Clinical observation of ShenQi FuZheng Injection Combined with chemotherapy in the treatment of gastrointestinal adenocarcinoma. Sichuan traditional Chinese medicine. 2006,24(7):45-47. DOI: 10.3969/j. issn.1000-3649. 2006.07.024.
20. Xin Ming, Wang Jinhua, Zhou Chunyu, et al. Clinical observation of ShenQi FuZheng Injection Combined with chemotherapy in the treatment of malignant tumor of digestive tract. Chinese Journal of integrated traditional Chinese and Western medicine. 1998,18(11):658. DOI:10. 3321/j.issn:1003-5370.1998.11.006.
21. Li Naiqing. Clinical observation and basic research on treatment of digestive tract malignant tumor with ShenQi FuZheng Injection Combined with chemotherapy. Journal of medical research. 1998(11):17-17.
22. ChengHuijun,Zhu Xia, Ge Hong. ShenQi FuZheng Injection Combined with chemotherapy in the treatment of digestive tract tumors. Journal of medical forum. 2006, 27(15):40-41. DOI :10.3969/j.issn.1672-3422.2006.15.023.
23. Sun Daxing,Ji Chunlian, QiuWeiyan, ShenHandeng. ShenQi FuZheng Injection Combined with chemotherapy in the treatment of advanced gastrointestinal tumors. Zhejiang Journal of integrated traditional Chinese and Western medicine. 2002,12(5): 284-285. DOI: 10. 3969/j.issn.1005-4561.2002.05.010.
24. Zhu Xia,Han Huijie. ShenQi FuZheng Injection Combined with chemotherapy in the treatment of advanced malignant tumor of digestive tract.Journal of Medical Forum. 2011(22):161-162.
25. Yang Yan. Attenuation effect of ShenQi FuZheng Injection in chemotherapy of gastrointestinal cancer. Gansu traditional Chinese medicine. 2007,20(7):38-39. DOI: 10. 3969/j.issn.1004-6852.2007.07.020.
26. Lu Dehong. Clinical observation of ShenQi FuZheng Injection in the treatment of upper digestive tract malignant tumor. Chinese and foreign health abstracts. 2011,08(13):268. DOI :10.3969/j.issn.1672-5085.2011.13.254.
