# Supplementary material for: ShenQi FuZheng Injection combined with chemotherapy in the treatment of colorectal cancer: A meta-analysis
Source: PLoS One. 2017 Sep 27;12(9):e0185254. doi: 10.1371/journal.pone.0185254 (PMC5617195; doi:10.1371/journal.pone.0185254)
Supplement: S2 File — (DOC) [file pone.0185254.s003.doc]

**S2_File: search strategy**

**pubmed**

#1 ((((((((((((((((Neoplasms, Colorectal[Title/Abstract]) OR Colorectal Neoplasm[Title/Abstract]) OR Neoplasm, Colorectal[Title/Abstract]) OR Colorectal Tumors[Title/Abstract]) OR Colorectal Tumor[Title/Abstract]) OR Tumor, Colorectal[Title/Abstract]) OR Tumors, Colorectal[Title/Abstract]) OR Colorectal Carcinoma[Title/Abstract]) OR Carcinoma, Colorectal[Title/Abstract]) OR Carcinomas, Colorectal[Title/Abstract]) OR Colorectal Carcinomas[Title/Abstract]) OR Colorectal Cancer[Title/Abstract]) OR Cancer, Colorectal[Title/Abstract]) OR Cancers, Colorectal[Title/Abstract]) OR Colorectal Cancers[Title/Abstract])) OR "Colorectal Neoplasms"[Mesh]

#2 ((shenqi fuzheng[Title/Abstract]) OR shenqifuzheng[Title/Abstract]) OR SFI[Title/Abstract]

窗体底端

#3 ("RCT*" OR "randomized controlled trial" OR "Randomized Controlled Trial" [Publication Type] OR "Randomized Controlled Trials as Topic"[Mesh] OR "Controlled Clinical Trial" [Publication Type])

窗体底端

#4 #1 and #2 and #3

**Embase**

PICOS search

#1 'colorectal cancer'/exp OR 'colorectal tumor'/exp

#2 'shenqi fuzheng injection'/exp OR sfi

#3 'chemotherapy'/exp AND 'randomized controlled trial'/exp

#4 #1 AND #2 AND #3

**Cochrane library**

#1 MeSH descriptor: [Randomized Controlled Trial] explode all trees

#2 MeSH descriptor: [Randomized Controlled Trials as Topic] explode all trees

#3 MeSH descriptor: [Randomized Controlled Trial] explode all trees

#4 MeSH descriptor: [Controlled Clinical Trial] explode all trees

#5 MeSH descriptor: [Controlled Clinical Trials as Topic] explode all trees

#6 #1 or #2 or #3 or #4 or #5

#7 窗体顶端

窗体顶端

7#&窗体底端

#7 MeSH descriptor: [Colorectal Neoplasms] explode all trees

#8 "colorectal cancer":ti,ab,kw or colorectal cancers:ti,ab,kw or colorectal tumor:ti,ab,kw or colorectal tumors:ti,ab,kw (Word variations have been searched)

#9 #7 or #8

#10 shenqi fuzheng:ti,ab,kw or shenqifuzheng:ti,ab,kw or "SFI":ti,ab,kw (Word variations have been searched)

#11 #6 and #9 and #10

窗体底端

**CNKI(Search in All Fields). Consider the following:**

#1 参芪扶正

#2 结直肠癌 or 结肠癌 or直肠癌 or 直肠肿瘤or 结肠肿瘤 or 结直肠肿瘤

#3 #1 and #2

**WangFang Date (Search in All Fields). Consider the following:**

#1 参芪扶正

#2 结直肠癌 or 结肠癌 or直肠癌 or 直肠肿瘤or 结肠肿瘤 or 结直肠肿瘤

#3 #1 and #2

**VIP (Search in All Fields). Consider the following:**

#1 参芪扶正

#2 结直肠癌 or 结肠癌 or直肠癌 or 直肠肿瘤or 结肠肿瘤 or 结直肠肿瘤

#3 #1 and #2

**SinoMed (Search in All Fields). Consider the following:**

#1 结直肠肿瘤

#2 结直肠癌

#3 结肠癌

#4直肠癌

#5直肠肿瘤

#6结肠肿瘤

#7 #1 or #2 or #3 or #4 or #5 or #6

#8参芪扶正

#9 #7 and #8
